# Supplementary material for: Acceptability and appropriateness of a risk-tailored organised melanoma screening program: Qualitative interviews with key informants
Source: PLoS One. 2023 Dec 13;18(12):e0287591. doi: 10.1371/journal.pone.0287591 (PMC10718433; doi:10.1371/journal.pone.0287591)
Supplement: S1 File — (PDF) [file pone.0287591.s001.pdf]

S1 File. Interview guide: Risk-tailored melanoma screening study

Script for interviewer: This will be spoken by the interviewer

For consistency, I'd like to share a definition of screening.

The purpose of screening is to identify people in an apparently healthy population (no signs or symptoms) who are at higher risk of a health problem (in this case melanoma) so that an early intervention or treatment can be offered. Unlike opportunistic early detection, an organised screening program typically involves offering screening to all people in an eligible group, usually defined by gender and age, and includes invitation letters and reminders, a clear referral system for management and follow-up, quality assurance measures and is publicly funded.

Experience with melanoma early detection
Clinicians, researchers, policy makers:<br>1. *To start with, can you tell me a bit about your current role and who you work with in this field?*<br>*OR*<br>*Consumers and consumer advocates:*<br>1. *To start with, can you tell me a bit about your own experience with melanoma and your current role/work in this area?*<br><br>2. *What do you see is the situation for early detection of melanoma in Australia at the moment?*<br><br>3. *What do you think about changing our current approach to melanoma early detection?*<br><br>4. *Do you think there are any factors hindering a change to the current melanoma early detection model in Australia?*<br><br>*Clinicians:*<br>5. *How does melanoma early detection currently fit into your clinical practice?*
An organised national screening program for melanoma
All participants:<br>7. *At the start of the interview, I mentioned the possibility of an organised national screening program for melanoma, similar to the programs we have for breast, cervical and colorectal cancers. What would an organised screening program for melanoma look like?*<br><br>*Clinicians, researchers, policy makers:*<br>8. *How do you see an organised screening program for melanoma fitting into clinical practice?*<br><br>*All participants:*<br>9. *Overall, how acceptable to [insert group they represent] do you think an organised screening program for melanoma would be?*<br><br>10. *How do you think the Australian community would feel about an organised melanoma screening program?*

11. *What do you think community preferences would be around a national screening program?*

A risk-tailored organised national screening program for melanoma – potential approaches

All participants:

I'd now like to ask your thoughts about a risk-tailored approach to a national organised melanoma screening program. For tailored screening we might use a range of risk factors in addition to age and family history such as lifestyle, environment and personal genomic information to provide tailored screening advice, rather than the one-size-fits all approach in our current programs. For example, people at higher risk would be recommended to receive more frequent skin checks and those at lower risk might be recommended to have few or no skin checks.

12. *What are your thoughts on a risk-tailored approach to organised melanoma screening?*
13. *If the organised screening program resulted in a change in existing screening intervals or frequency for some people (depending on personal risk level), such as an increase or decrease in skin checks, or perhaps not screening at all, how acceptable do you think this would be to the Australian community?*
14. *How do you think the risk assessment process should occur?*

Specific approaches to rolling out an organised national screening program for melanoma

All participants:

15. *Do you think there is a role for novel technologies such as apps, total body photography, teledermatology or artificial intelligence to be used to support an organised screening program?*
16. *How do you think an organised screening program for melanoma should be funded?*

Primary prevention and the potential for a national screening program

All participants:

17. *What role should prevention strategies, for example encouraging people to wear sunscreen and slip, slop, slap, have in relation to an organised screening program for melanoma?*

Policy and health system considerations

All participants:

18. *In the final part of the interview, I'd like to turn your thoughts to the broader health system and policy considerations regarding melanoma. Overall, what would you say is the most important factor in ensuring the successful implementation of an organised melanoma screening program.*
19. What do think would be the evidence base needed to support such a program?
20. *Overall, what would be the most significant barriers to the successful implementation of an organised screening program for melanoma?*

Clinicians, researchers and policy makers:

21. *Do you foresee any conflicts of interest arising in relation to an organised screening program?*

22. *What key clinical or performance indicators do you think should be measured to monitor the quality of a melanoma screening program or for people having skin checks?*

23. *To finish up, do you have any thoughts on the steps towards or the feasible timing for implementation of such an organised melanoma screening program?*

All participants:

24. *Is there anything else that you'd like to raise?*

Thank you again for speaking with me today and sharing your views. Would you be interested in completing an online questionnaire about this topic?

As you might understand we are trying to maximise our recruitment efforts on this study and are looking to get a broad scope of clinician opinions on these changes. Are there any colleagues you can think of who might be interested in participating in this study? (If yes, ask for contact details or send follow-up email)
