# Supplementary material for: Acceptability and appropriateness of a risk-tailored organised melanoma screening program: Qualitative interviews with key informants
Source: PLoS One. 2023 Dec 13;18(12):e0287591. doi: 10.1371/journal.pone.0287591 (PMC10718433; doi:10.1371/journal.pone.0287591)
Supplement: S1 Table — (PDF) [file pone.0287591.s002.pdf]

S1 Table. Coding framework with domains, relevant determinants, definitions and additional supporting quotes

Determinant	Definition	Supporting quotes
**Domain: Guideline Factors**		
Quality of evidence supporting the recommendation	How confident we are in the estimates of effects	*"I think we'll have enough evidence to show that that is something that should proceed. I hope that the Commonwealth feels the same" (P32, Researcher)*<br>*And also off the opportunistic screening program that is happening, there is already a lot of screening ongoing and so that would make it difficult to have a true control group that would allow unbiased comparison of the value of the screening program" (P25, Researcher)*
Feasibility	The extent to which the recommended clinical intervention is practical (for health professional and in this setting)]	*"So in my own clinical practice (skin cancer clinic in primary care), I see a lot of referred patients, a lot of GPs refer patients to me and not just for a skin check, but for actually what is quite sophisticated management of skin cancer and skin cancer risk" (P34, GP)*
Compatibility	The extent to which the recommended clinical intervention fits with current practices	*"my view is that what's essentially primary and secondary prevention, screening is a secondary prevention, have to sit hand in hand... And it would be the mechanism by which public messaging could be encapsulated in whatever it is that's developed" (P1, Policy expert)*
**Domain: Individual Health Professional Factors**		
Skills needed to adhere	The extent to which the targeted healthcare professionals have skills to adhere	*"even with skin cancer clinics, there are a lot of skin cancer clinics with questionable skill.. So yeah, that'd be my only concern, is who's doing the screening"(P16, GP)*
Agreement with recommendation	The extent to which the targeted healthcare professionals agree with the recommendation	*"So risk adjustment is essential, absolutely essential. And if we were talking about a melanoma surveillance program, which would obviously be funded through public funds, you would have to go risk stratified because you've got to allocate limited resources accordingly" (P34, GP)*<br>*"Structuring (a formal screening program) more the 'high-risk' group, but not taking anything away from those lower-risk patients who still have those options at the moment" (P26, GP)*<br>*"I wouldn't want people at low risk ending up with no screening at some point. Like even the people that we think are at low risk, we still pick up – they can still end up with a melanoma and their first melanoma" (P16, GP)*
Nature of the (professional) behaviour	Characteristics of the behaviour, including: frequency of performance for a patient, frequency of performance for a population of patients, the degree of habit or automaticity, whether it is within a sequence of other behaviours that have to be performed, and whether it is performed	*"in a perfect world, I think you would have these online risk calculators, which of course exist already. You'd ensure that populations are aware that they could do the self-assessment at home, but also you would have systems within general practice where the practice nurse could be doing the risk assessment and then those who met the risk cut-off could be referred on for a skin check" (P30, Researcher)*

	by one person or by different people	
**Domain: Patient Factors**		
Patient preferences	Patients' values in relationship to professional values or those in the recommendation	*"I think it'd be fantastic. I think they (consumers) would value it immensely, one, because it gives them knowledge that they didn't have and they've gone through a process to give them that information and to give them a rating or awareness, but two it also gives them the confidence" (P13, Consumer)*
Patient behaviour	Patient behaviours that motivate or demotivate adherence with the recommendation	*"The people who are not high risk, I think they still have to have an avenue to go. I mean there's spontaneous melanomas, they still have to be able to be checked. As long as they know that that's still open to them, I think it's not too bad. It's really in the marketing, I think, how you market that whole strategy is critical" (P21, Consumer)*
**Domain: Professional Interactions**		
Communication and influence	The extent to which the targeted healthcare professionals' adherence is influenced by professional opinions and communication	*"it needs to engage primary care in ways that they're still not engaged with bowel or breast and at times, cervical cancer" (P8, Policy expert)*
**Domain: Incentives and Resources**		
Financial incentives and disincentives	The extent to which patients, individual health professionals and organisations have financial incentives and disincentives	*"the biggest incentive for GPs to start doing, for example, screening programs or something along those lines is probably you incentivise it and at the moment they do healthcare plans, which there's good monetary incentive. It can become part of a health management plan, at least a skin check is a simple thing to add on to those things" (P16, GP)*
Assistance for clinicians	The extent to which clinicians have the assistance they need to adhere	*"I see that as a fairly useful thing (novel technologies) to reduce the burden on healthcare, on medical staff actually, yes" (P35, Dermatologist)*
**Domain: Capacity for Organisational Change**		
Relative strength of supporters and opponents	The mandate, authority and accountability for making necessary changes	*"I think the skin cancer clinics who are obviously a major part of this quasi skin cancer screening program that we have, they are variable as to how they assess risk. But I think that's part of the implementation really, is if you're going to be billing Medicare for a skin check, then as part of a program you would need to demonstrate what their risk score was and which risk tool had been used to assess that risk and that would be tied to the MBS billing. It's probably the only way you could control it. (P30, Researcher)*
Monitoring and feedback	The extent to which monitoring and feedback are needed at organisational level and available to sustain necessary changes (including evaluations of improvement programs)	*"In terms of the patients' point of view, satisfaction, acceptability, trust, there's nothing else really that I would say in terms of process outcomes. In the long term, you would want to see, as you've said, stage of melanoma, fewer melanomas being diagnosed for late stage and improved survival for melanoma, or rather, reduced mortality from melanoma" (P32, Researcher)*
Priority of necessary change	The relative priority given to making necessary changes	*"I mean my personal preference is on primary prevention, but that is not going to be 100 per cent effective ever. And so then we need early detection to pick melanomas up early" (P24, Researcher)*

Domain: Social, Political and Legal Factors		
Economic constraints on the health care budget	Limits on the total healthcare budget or its growth	*"I think it was designed correctly, it would have to be cheaper than the current state. Because you would hope that it would reduce the overservicing that is currently happening" (P35, Dermatologist)*<br>*"Its going to require really robust modelling and hard, cold figures to convince government that this is an investment that they should make" (P8, Policy expert)*<br>*"we also have to do the economic analysis to enable us to talk to governments in a way in which they would appreciate, which is around the return on investment, it is around the wasted dollars that they're currently investing and presenting a picture to them where opportunities are to save money, but also save lives" (P2, Policy expert)*
Payer or funder policies	The extent to which payer or funder policies may affect implementation of necessary changes	*"It's always that challenge as well, the state versus federal and where you've got hospital services that are funded by state funding and you've got obviously all primary care services are funded federally through Medicare and how that overlap often occurs with a screening program is sometimes interesting" (P26, GP)*
Influential people	The extent to which influential people may affect implementation of necessary changes	*"Structured formal screening program probably leads to a move away from potential conflicts of interest as long as its collaborative" (P26, GP)*
