# Supplementary material for: Acceptability and appropriateness of a risk-tailored organised melanoma screening program: Qualitative interviews with key informants
Source: PLoS One. 2023 Dec 13;18(12):e0287591. doi: 10.1371/journal.pone.0287591 (PMC10718433; doi:10.1371/journal.pone.0287591)
Supplement: Attachment — Submitted filename: Comment author PLOS ONE Qualitative interviews with key informants.pdf [file pone.0287591.s003.pdf]

Acceptability and appropriateness of a risk-tailored organised melanoma screening program: Qualitative interviews with key informants

Comments to the Authors

Congratulation on the submitted manuscript. The topic is timely and will be of interest to the readers of the journal. However, few changes are suggested to improve the clarity of this manuscript.
I have several recommendations and questions about the manuscript.

No	Page, Para	Descriptions of item	Comment
1.		Abstract	Suggest changing the pronoun word "we" to "researcher"<br>Please explain the inclusion and exclusion criteria in the method.<br>The author should specify what was the 'valuable information' in the conclusion and briefly describe the importance of the 'valuable information"
	4,83	Introduction<br><br>Risk-tailored screening, also referred to as 'risk-stratified screening' or 'targeted screening', tailors screening (e.g. eligibility,frequency, intervals, type of test) to individual risk rather than the mostly one-size-fits-all approach of organised population screening programs.	The introduction section has a clear statement demonstrating that the focus of the study. The problem definition is stated clearly.<br><br>Please add the reference<br>Please add citations when necessary.
	5,113	This qualitative study aimed to explore the acceptability and appropriateness of population based, organised risk-tailored melanoma screening from the perspective of key informants.	Please harmonize the statement

	6,120	Methodology	I would like to suggest to the author provide more information on the methodology of the study such as:<br>-How about the sample size selected?<br>-The total number of respondents from four expert groups?<br>-The inclusion and exclusion criteria?<br>-The semi-structured interview protocol process consisted of what questions?
	6,133	Ethical consideration	Please add the reference number or approval code.
	9,198<br><br><br><br>21,469	Table 1<br><br><br><br>Discussion	Please add the subtitle in the table<br>Example:<br>Frequency(n)<br><br>How do the findings add to the body of scientific knowledge on the particular issue?<br>Should highlight the implication of the results to health policy.
	26,588	Summary	Please make a subheading for summary
	27		Please state the source of the research funding including the grant name and number.
	27,610	References	Please add doi in the article's references.<br>Example:<br>Trager MH, Queen D, Samie FH, Carvajal RD, Bickers DR, Geskin LJ. Advances in Prevention and Surveillance of Cutaneous Malignancies. Am J Med. 2020 Apr;133(4):417-423. doi: 10.1016/j.amjmed.2019.10.008. Epub 2019 Nov 9. PMID: 31712100; PMCID: PMC7709483.
